# Accelerated sequences of 4D flow MRI using GRAPPA and compressed sensing: A comparison against conventional MRI and computational fluid dynamics

**DOI:** 10.1002/mrm.29404

**Published:** 2022-08-25

**Authors:** Morgane Garreau, Thomas Puiseux, Solenn Toupin, Daniel Giese, Simon Mendez, Franck Nicoud, Ramiro Moreno

**Affiliations:** ^1^ University of Montpellier, CNRS Montpellier France; ^2^ Spin Up, ALARA Group Strasbourg France; ^3^ I2MC, INSERM/UPS UMR 1297 Toulouse France; ^4^ Siemens Healthcare France Saint‐Denis France; ^5^ Magnetic Resonance, Siemens Healthcare GmbH Erlangen Germany; ^6^ ALARA Expertise, ALARA Group Strasbourg France

**Keywords:** 4D flow, compressed sensing, computational fluid dynamics (CFD), flow phantom

## Abstract

**Purpose:**

To evaluate hemodynamic markers obtained by accelerated GRAPPA (*R* = 2, 3, 4) and compressed sensing (*R* = 7.6) 4D flow MRI sequences under complex flow conditions.

**Methods:**

The accelerated 4D flow MRI scans were performed on a pulsatile flow phantom, along with a nonaccelerated fully sampled k‐space acquisition. Computational fluid dynamics simulations based on the experimentally measured flow fields were conducted for additional comparison. Voxel‐wise comparisons (Bland–Altman analysis, $L_{2}$‐norm metric), as well as nonderived quantities (velocity profiles, flow rates, and peak velocities), were used to compare the velocity fields obtained from the different modalities.

**Results:**

4D flow acquisitions and computational fluid dynamics depicted similar hemodynamic patterns. Voxel‐wise comparisons between the MRI scans highlighted larger discrepancies at the voxels located near the phantom's boundary walls. A trend for all MR scans to overestimate velocity profiles and peak velocities as compared to computational fluid dynamics was noticed in regions associated with high velocity or acceleration. However, good agreement for the flow rates was observed, and eddy‐current correction appeared essential for consistency of the flow rates measurements with respect to the principle of mass conservation.

**Conclusion:**

GRAPPA (*R* = 2, 3) and highly accelerated compressed sensing showed good agreement with the fully sampled acquisition. Yet, all 4D flow MRI scans were hampered by artifacts inherent to the phase‐contrast acquisition procedure. Computational fluid dynamics simulations are an interesting tool to assess these differences but are sensitive to modeling parameters.

## Abstract 2

Click here for author‐reader discussions

**Abbreviations**
CFD: computational fluid dynamics
CFD_HR: high‐resolution (“true”) computational fluid dynamics
CFD_LR: low‐resolution (downsampled) computational fluid dynamics
CS: compressed sensing
FS: fully sampled
G: GRAPPA (e.g., G3: GRAPPA R=3)
PC: phase‐contrast
VENC: velocity encoding

## 1 INTRODUCTION

2D phase‐contrast (PC) MRI is a well‐established blood flow measurement technique to evaluate cardiovascular disorders such as valvular diseases, aortopathies, or congenital heart diseases.^1^, ^2^ More recently, time‐resolved 3D PC imaging, referred to as *4D flow MRI*, has gained significant interest for its ability to provide in vivo quantification of blood flow dynamics inside a 3D volume over the cardiac cycle.^3^, ^4^ Whereas 2D PC imaging is operator‐dependent for plane positioning, 4D flow imaging provides a retrospective flow quantification at any location within the acquired volume. In addition to providing comprehensive velocity and vascular motion in a single scan, 4D flow MRI also opens access to advanced hemodynamic biomarkers such as wall shear stress (WSS),^5^ pulse wave velocity,^6^ or relative pressure.^7^ To this respect, 4D flow MRI has the potential to become a gold‐standard practice in clinical routine. However, the clinical applicability of this technology remains hampered by its inherently long scan duration, which is further worsened by respiratory gating techniques for motion compensation. Despite the use of parallel imaging techniques (e.g., GRAPPA and SENSE) with typical acceleration factors of 2–3, 4D flow scan times still range between 5 and 15 min. Therefore, alternative acceleration techniques have been developed over the years to further shorten 4D flow scan duration by exploiting spatiotemporal correlations: k‐t GRAPPA,^8^, ^9^ broad‐use linear acquisition speed‐up technique (k‐t BLAST),^10^ and non‐Cartesian acquisition sampling,^11^ to cite a few. However, these strategies are limited by long reconstruction times, mostly offline, making them hardly compatible with clinical workflows.

In the last years, a compressed sensing (CS) 4D flow framework has shown great potential for decreasing the scan time with a reconstruction performed inline in less than 5 min.^12^, ^13^ This performance was achieved using a k‐t accelerated Cartesian pulse sequence with a variable‐density phyllotaxis undersampling and L_1_‐regularized wavelet‐based reconstruction. Ma et al.^12^ first demonstrated the feasibility of this framework in vitro using a realistic aorta flow phantom with various CS acceleration factors, and for 20 healthy volunteers with a CS acceleration factor of *R* = 7.7. Pathrose et al.^13^ assessed the same framework on patients with aortic diseases with 3 different CS factors (*R* = 5.7, 7.7 and 10.2) compared to a GRAPPA‐accelerated sequence (*R* = 2). Both studies have consistently shown a significant underestimation of measured maximum velocity and flow within 10%–15%, as for derived parameters such as WSS. The higher the CS acceleration factor, the higher the underestimation. However, the factors leading to this underestimation are still not fully understood even though both studies suggest that spatiotemporal undersampling and regularization could be responsible for this trend. Moreover, whereas GRAPPA‐accelerated sequences are considered a clinical gold standard, they are expected to induce additional flow quantification errors as compared to fully sampled (FS) k‐space sequences.^14^ To characterize the nature of the errors, it is relevant to compare a CS‐accelerated sequence with a FS sequence, where no undersampling is involved. Also, standalone parameters such as the mass conservation can also be relevant to estimate the degree of discrepancies,^15^ with no need of reference measurement. Additionally, significant underestimations of WSS are generally observed, partly because of partial volume effects and low spatiotemporal resolution.^16^ Given the growing interests for evaluating the WSS clinically, substantial efforts are being undertaken to propose sophisticated reconstruction methods.^5^, ^17^, ^18^ However, whereas little attention is generally paid to assess the quality of the input velocity measurements, it is a prerequisite step to properly reconstruct the WSS.

Alternatively, the flow field can be predicted by coupling MRI measurements with computational fluid dynamics (CFD).^19^, ^20^, ^21^, ^22^ This approach bypasses the experimental limitations inherent to MRI acquisitions, such as spatiotemporal resolution or noise, while satisfying the fluid mechanics laws. CFD coupled to MRI has already proven capable of providing the flow fields with high fidelity under well‐controlled in vitro conditions,^23^, ^24^ whereas moderate correlations have been reported for patient‐specific MRI‐based simulations^25^, ^26^ or superresolution of 4D flow MRI using CFD^27^ for velocity and flow rates. Indeed, the choice of the CFD strategy is crucial to accurately predict the hemodynamics, particularly in such flow regimes where boundary conditions^28^ and turbulence models,^29^, ^30^ as well as numerical schemes,^31^ have shown to greatly influence the resulting flow field. In this context, CFD may be used as a third‐party modality, yet without being considered a ground truth, to confirm and quantify the discrepancies observed with 4D flow MRI.

The main objective of this study was to investigate the flow errors induced by GRAPPA‐ and CS‐accelerated 4D flow MRI sequences under complex flow conditions. The experiments were conducted on a previously designed pulsatile flow phantom for which the geometry yields flow patterns similar to the complex flow structures observed in vivo: recirculation, flow split, large‐scale transitioning turbulence features, etc. High correlation between nonaccelerated 4D flow MRI sequence and CFD was already demonstrated in this well‐controlled environment following appropriate postprocessing methods.^23^ In the present study, several 4D flow MRI scans with GRAPPA (*R* = 2, 3, 4, abbreviated respectively G2, G3 and G4 in the following) and CS (*R* = 7.6) accelerations were acquired and compared with a conventional full k‐space sampling sequence. Moreover, a high‐fidelity CFD solution fed by boundary conditions compatible with the measured flow field was generated and used as a supplementary means to characterize the flow measurement errors.

## 2 METHODS

The phantom experiment, along with the CFD simulation process, have been described previously in Puiseux et al.,^23^ where more details are available. A summary is given hereafter.

### 2.1 Phantom experimental setup

**FIGURE 1:**
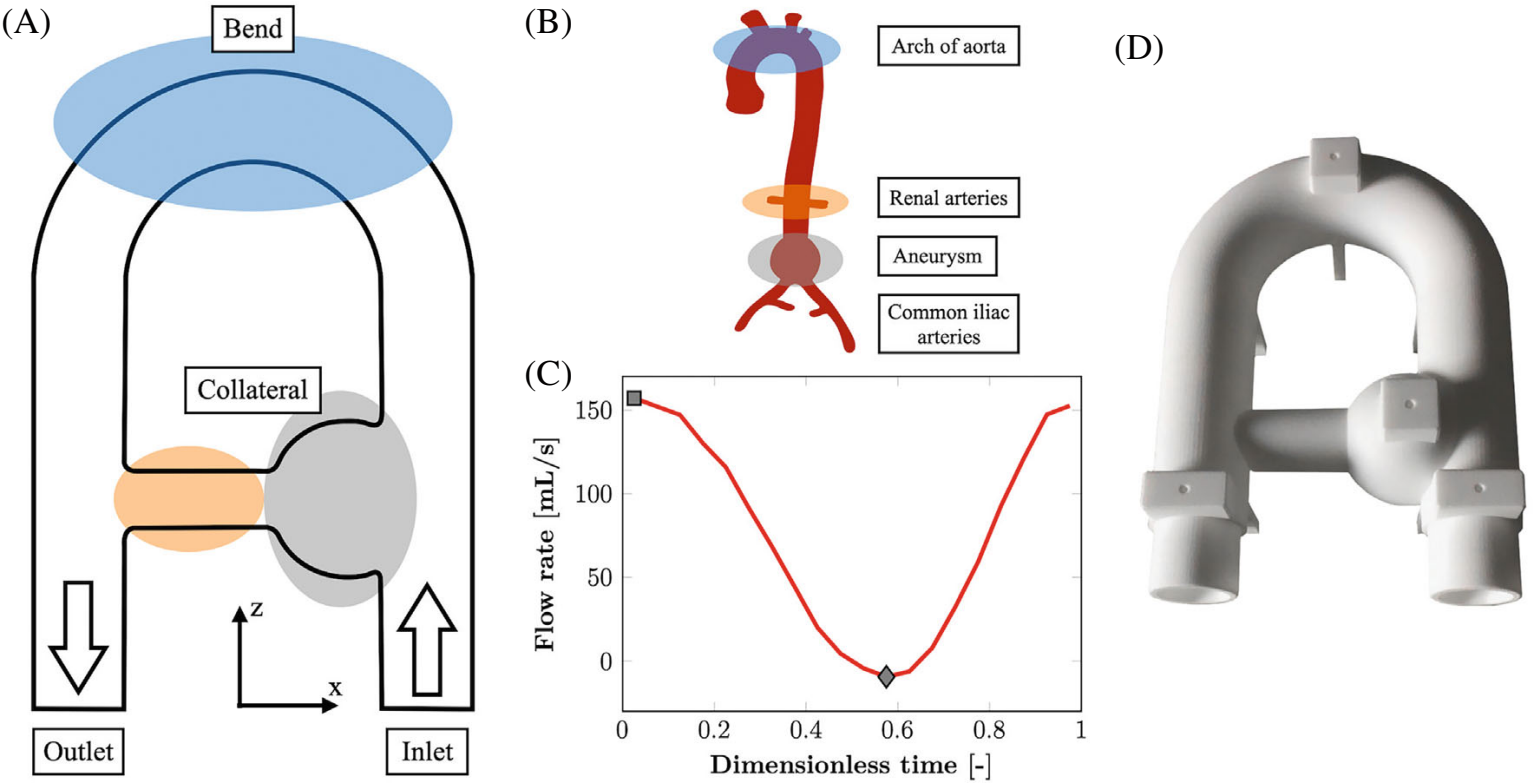
Phantom geometry and flow regime. (A) Sketch of the flow phantom. (B) Analogy with cardiovascular system. (C) Flow rate at the inlet for the fully sampled 4D flow acquisition corrected according to the postprocessing procedure detailed in the corresponding section. The square and the diamond correspond to peak systole and end diastole, respectively. (D) Photograph of the 3D‐printed flow phantom.

A rigid flow phantom made up with nylon was designed to reproduce complex flow patterns as reported in the cardiovascular system (Figure 1A,B). The phantom was embedded into a silicone bath to increase the SNR and connected to a programmable pump (CardioFlow 5000 MR, Shelley Medical Imaging Technologies, London, Ontario, Canada) installed outside the 5 Gauss line via pipes. The pulsatile flow rate delivered by the pump (Figure 1C) was measured by means of an ultrasonic flowmeter (UF25B100 Cynergy3 components Ltd, Wimborne, Dorset, UK) placed upstream of the entrance of the phantom. A schematic representation of this experimental setup can be found in Ref. ^23^ By analogy with the cardiac cycle, the times of maximum and minimum flow rates are referred to as peak systole and end diastole, respectively. A blood‐mimicking fluid was supplied to the phantom circuit with kinematic viscosity $\nu =4.02\times 10^{-6}\;$ m^2^/s, density ρ=1020 kg/m^3^, and relaxation times $T_{1}=0.85$ s and $T_{2}=0.17$ s at 1.5 Tesla.

### 2.2 MRI data acquisitions

MRI data were obtained thanks to a 1.5 Tesla Siemens Magnetom Sola (Siemens Healthcare, Erlangen, Germany) using a prototype 4D flow MRI sequence. The sequence was retrospectively gated using a simple 4‐point velocity‐encoding scheme.^32^ The electrocardiogram trigger needed for gating was simulated by means of an MRI‐compatible fake finger (MR Finger, Shelley Medical Imaging Technologies, London, Ontario, Canada). It delivered an infrared signal synchronized with the pump waveform cycle and interpreted as an electrocardiogram signal via the peripheral pulse unit of the MRI scanner. Thereby, what is referred to as cardiac cycle in the following is the pump cycle, for which the averaged duration is close to 1 s. A FS sequence and several GRAPPA (*R* = 2, 3, 4) and CS (*R* = 7.6) accelerated sequences were acquired. The acquisition and reconstruction frameworks used for the latter pulse sequence can be found in Ref.^12^ The main scan parameters, among which are TE, TR, and 3D velocity encoding (VENC), are listed in Table 1.

**TABLE 1:** Imaging parameters

Imaging technique	FS	G			CS
Acceleration rate, *R*	–	2	3	4	7.6
Scan time (min:s)	42:40	21:20	14:40	10:40	5:35
FOV (mm^3^)	256 × 256 × 72				
Acquired voxel size (mm^3^)	2 × 2 × 2				
Receiver bandwidth (Hz/pixel)	383				
Flip angle (°)	7				
VENC (x, y, z) (cm/s)	70‐20‐70				
TE (ms)	4.15				3.70
TR (ms)	6.48				6.04
Temporal resolution (ms)	51.8				48.3
Number of reconstructed cardiac phases	20				25

CS, compressed sensing; FS, fully sampled; G, GRAPPA; VENC, velocity encoding.

### 2.3 CFD simulations

The simulations were carried out using YALES2BIO (https://imag.umontpellier.fr/∼yales2bio/), an in‐house large eddy simulation solver using finite‐volume method and designed to perform numerical simulations of blood flows in complex geometries.^33^ In large eddy simulation, the largest turbulent scales are explicitly resolved as a solution of the low‐pass filtered Navier–Stokes equations, whereas the subgrid scales are modeled. Thereby, the computational costs are reduced in comparison with a direct numerical simulation in which the whole range of spatial and temporal scales of turbulence must be resolved. Due to the complex geometry used in this study to induce large scale fluctuations, as well as the flow regime being in the laminar–turbulent transition, the large eddy simulation strategy was preferred to the Reynolds‐averaged Navier–Stokes modeling, where all the scales are averaged and the entire turbulence spectrum is modeled.

The fluid was modeled as incompressible Newtonian with the already mentioned mechanical properties. A tetrahedral‐based mesh of the phantom with a characteristic cell size of 0.7 mm was generated with Gambit 2.4.6 (ANSYS, Inc., Canonsburg, PA) and used to solve the incompressible Navier–Stokes Equations. A zero‐pressure condition was prescribed at the outlet, whereas a no‐slip condition was imposed at the solid boundaries. Regarding the inlet boundary condition, a pixel‐based inflow was derived from the MRI acquisition velocity field, which was corrected according to the postprocessing procedure detailed in the following section. Hence, 1 CFD simulation by MR acquisition was generated.

The mesh cell size was defined using a mesh sensitivity analysis. Four different tetrahedral‐based meshes were investigated based on the inlet provided by the FS acquisition: a coarse one with 622 thousand cells (cell size = 1.3 mm), a medium one with 1284 thousand cells (cell size = 1.0 mm), a fine one with 3812 thousand cells (cell size = 0.7 mm), and a finer one with 27 million cells (cell size = 0.35 mm). The relative error on the phase‐averaged velocity magnitude (cf. definition in the section 2.4) between the 2 latter meshes came to 0.9% of the maximum velocity magnitude found for the finer mesh. Thereby, the velocity field was considered to be spatially converged and independent of the spatial resolution for the fine mesh. More details on the numerical accuracy (sensitivity analysis on mesh, phase‐averaging, and turbulence resolution) can be found in Ref.^34^

### 2.4 Postprocessing

**FIGURE 2:**
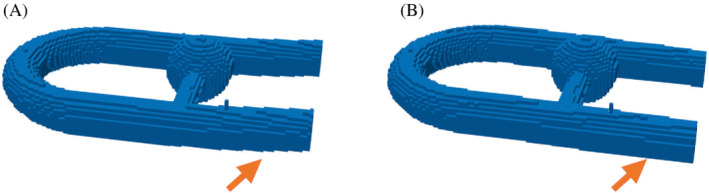
Distortion correction presented as a threshold on the image magnitude. (A) Before distortion correction. (B) After correction. The correction is applied on the whole phantom, but its effect is more noticeable at the inlet and outlet, as highlighted by the orange arrows.

The 4D flow data went through an in‐house postprocessing procedure programmed in Python (http://www.python.org, version 3.8.2). Maxwell terms,^35^ as well as in‐plane distortions induced by the nonlinearities of the magnetic gradient field, were corrected within the reconstruction process of the MRI system. An additional correction in the through‐plane direction based on the knowledge of the phantom geometry was performed. To do so, the distorted volume was segmented thanks to a threshold on the magnitude images, and a second‐order polynomial fit was performed on the coordinates of the centerline throughout the parallel branches of the main pipe. The fit found was used to relocate the voxels position along the through‐plane direction such that the centerline lies in the coronal plane. Note that every voxel underwent this correction, but the further away from the isocenter (localized above the collateral), the greater the position shift (see Figure 2). Further corrections consisted in noise masking and phase unwrapping. A presegmentation of the flow volume was obtained by thresholding the image magnitude averaged over time for registration purposes only. The resulting presegmented volume was registered onto the computational model thanks to an iterative closest point algorithm. Finally, an eddy current correction was implemented according to Lorenz et al. method,^36^ based on the assumption that the velocity field measured in static regions should be exactly zero. The silicone bath surrounding the flow phantom was used for this purpose. After segmentation, the voxels belonging to this static region were fitted with a linear function of the space coordinates using a least squares method. This was done for each time frame and velocity direction. The corrected velocity field was obtained by subtraction of the fitted plane. The velocity field resulting from the application of all the corrections described above is referred to as the corrected MR in what follows. All MR acquisitions underwent these same postprocessing steps.

To compare MRI acquisitions with CFD simulations, the latter went through phase‐averaging and downsampling following the procedure described in Puiseux et al.^23^ The reason for phase‐averaging the CFD velocity field is that there are cycle‐to‐cycle fluctuations when simulating such an unsteady flow lying in the laminar–turbulent transition.^37^ Furthermore, the MR signal is also acquired over numerous cardiac cycles. Thereby, 40 cardiac cycles were simulated. The first 10 cycles were taken out of the comparison to cancel the effect of the initial condition (zero velocity condition). The resulting CFD velocity field, phase‐averaged over the last 30 simulated cardiac cycles, was then downsampled on an image grid with the same spatial resolution as the MRI acquisitions. This low‐resolution field is referred to as *CFD_LR* thereafter, whereas *CFD_HR* refers to the “true” CFD.

Because both the CFD_LR and corrected MR velocity fields were finally expressed on the same grid, and the phantom geometry is a priori known, the segmentation of the flow volume was obtained by thresholding the CFD_LR velocity magnitude averaged over time.

### 2.5 Comparison methods

**FIGURE 3:**
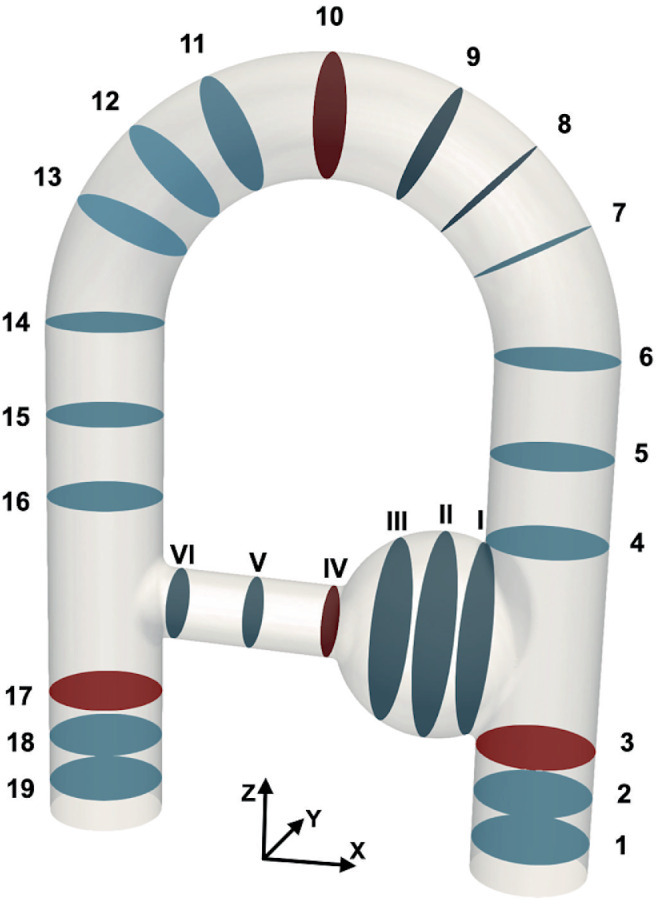
Labeling of the planes used to analyze the differences between the velocity fields measured with the different modalities. The planes in the main duct are numbered 1–19 from inlet to outlet, and the planes in the collateral duct are numbered I to VI. The planes in red are highlighted for better readability of Figure 9.

The different MRI modalities were quantitively compared by conducting Bland–Altman analysis to evaluate the agreement (bias ± SD) between the pointwise velocity fields obtained from FS and accelerated MR sequences, as well as with the CFD_LR fields. Furthermore, the $L_{2}$ ‐norm (also called Euclidean distance) was used as a metric to measure the pointwise similarity between the velocity fields obtained from the different methods. The normalized $L_{2}$ ‐norm calculated at each node position x and at each time instant t for 2 fields A and B is expressed as:

(1) $$L_{2}\left(x,t\right)=\frac{\sqrt{{\left(u_{A}-u_{B}\right)}^{2}+{\left(v_{A}-v_{B}\right)}^{2}+{\left(w_{A}-w_{B}\right)}^{2}}}{\bar{\left|\left|u_{\text{bulk}}\right|\right|}}$$

where u=(u,v,w) is the velocity vector associated to the node at the position x , and $\bar{\left|\left|u_{\text{bulk}}\right|\right|}=0.144$ m/s is the time‐averaged bulk velocity magnitude measured at the inlet surface for the FS acquisition. Velocity profiles, flow rates, and peak velocities were studied in 19 planes along the main duct and 6 along the collateral duct, numbered respectively 1 to 19 and I to VI from the inlet side to the outlet side (see Figure 3). Some comparisons are said to be performed on all voxels, whereas others are done on inner voxels only. *All voxels* designates all the voxels segmented from the flow volume (cf. section 2.4) with edge voxels straddling the phantom walls included, whereas *inner voxels* corresponds to the voxels strictly included in the phantom without the edge voxels. For each MRI modality, the segmentation includes about 53,500 voxels, against around 26,800 inner voxels.

## 3 RESULTS

Scan times achieved for the FS; G2; G3; G4; and CS 4D flow MRI are 42:40, 21:20, 14:40, 10:40, and 5:35 min, respectively. Investigating whether this strong acceleration comes with a measurable degradation of the quality of the results is the objective of the following subsections.

### 3.1 Flow structures and velocity profiles

**FIGURE 4:**
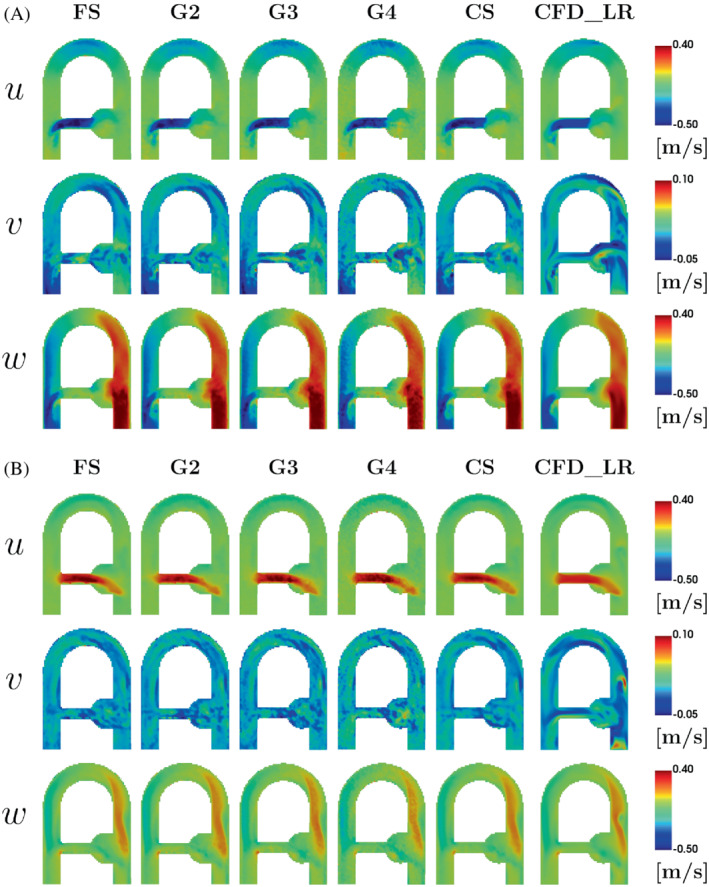
Velocity fields in the coronal plane at (A) peak systole and (B) end diastole (cf. Figure 1C). The rows represent the velocity components **u** = (*u*, *v*, *w*). The columns show the low‐resolution downsampled CFD (CFD_LR) and MRI acquisitions. CFD, computational fluid dynamics; CFD_LR, computational fluid dynamics–low‐resolution; CS, compressed sensing; FS, fully sampled; G, GRAPPA.

**FIGURE 5:**
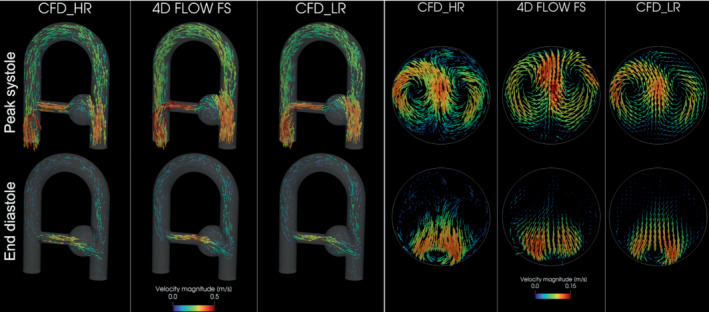
3D vector‐based visualization of the FS acquisition and the CFD simulations at peak systole (on the top row) and end diastole (on the bottom row). The whole phantom is displayed on the left‐hand side, whereas a slice in the middle of the aneurysm‐like region (corresponding to the slice II in Figure 3) is presented on the right‐hand side. The vectors are scaled by velocity magnitude.

As presented in Figure 4, the main flow structures are similarly captured by all sequences and by CFD_LR for the velocity components u and w both at peak systole and end diastole, whereas more disturbed results are found for the low velocity field v. From now on, the results presented in this study will focus on the FS, G3, and CS acquisitions for the sake of clarity. Indeed, because it can already be visually noticed in Figure 4, the G4 velocity field appears noisier in comparison to the other sequences, and the quantitative comparisons lead to poor outcomes for this acquisition. Good results are found for G2, but due to its long acquisition time the preference has been to present the comparisons with G3. To further motivate this choice, the global $L_{2}$‐norm over all voxels is provided as Supporting Information Figure S1, which is available online. The flow structures are further apprehended thanks to vector‐based visualization of the MRI and corresponding CFD_HR and CFD_LR. Figure 5 displays the velocity vector field in the whole phantom, as well at the middle plane of the aneurysm‐like region for the FS acquisition along with the corresponding CFD_HR and CFD_LR fields. Although the CFD_LR partially mimics the MRI acquisition process, the flow structures localizations are well reproduced. At peak systole, counterrotating vortices are observed for both the FS acquisition and the CFD simulations, although localized higher in the slice for the MRI as compared to CFD simulations. Videos of both kinds of vector visualization for the FS, G3, and CS acquisitions, along with the corresponding CFD simulations, are available as Supporting Information (Supporting Information Videos [Link], [Link]).

**FIGURE 6:**
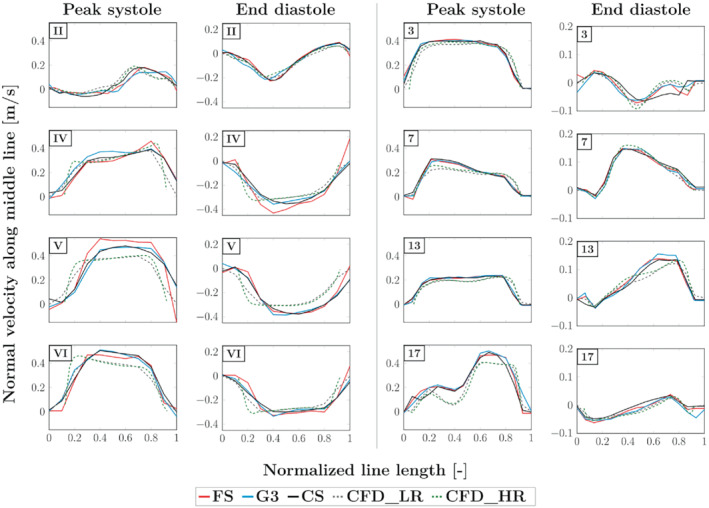
Velocity profiles along lines located in the coronal plane passing through the middle of the phantom. The velocity displayed corresponds to the projection onto the normal of the planes perpendicular to the ducts, referenced as in Figure 3 (the corresponding slice number is indicated in the corner of each graph).

The velocity profiles for the 3 MRI methods are presented in Figure 6, along with those obtained from the CFD simulations. Whereas velocity profiles are globally in good agreement for all MRI modalities, the MRI velocity tends to overestimate the CFD one, especially in regions and at time instants of high velocity or acceleration (e.g., planes V and 17 at peak systole in Figure 6). Moreover, a lateral shift of the MR profiles with respect to the CFD profiles is noticeable in the collateral duct. Finally, a small overestimation of the FS as compared to CS and G3 (14.7% and 12.3% for peak velocity, respectively) can be observed at peak systole in plane V.

### 3.2 Statistical comparison

**FIGURE 7:**
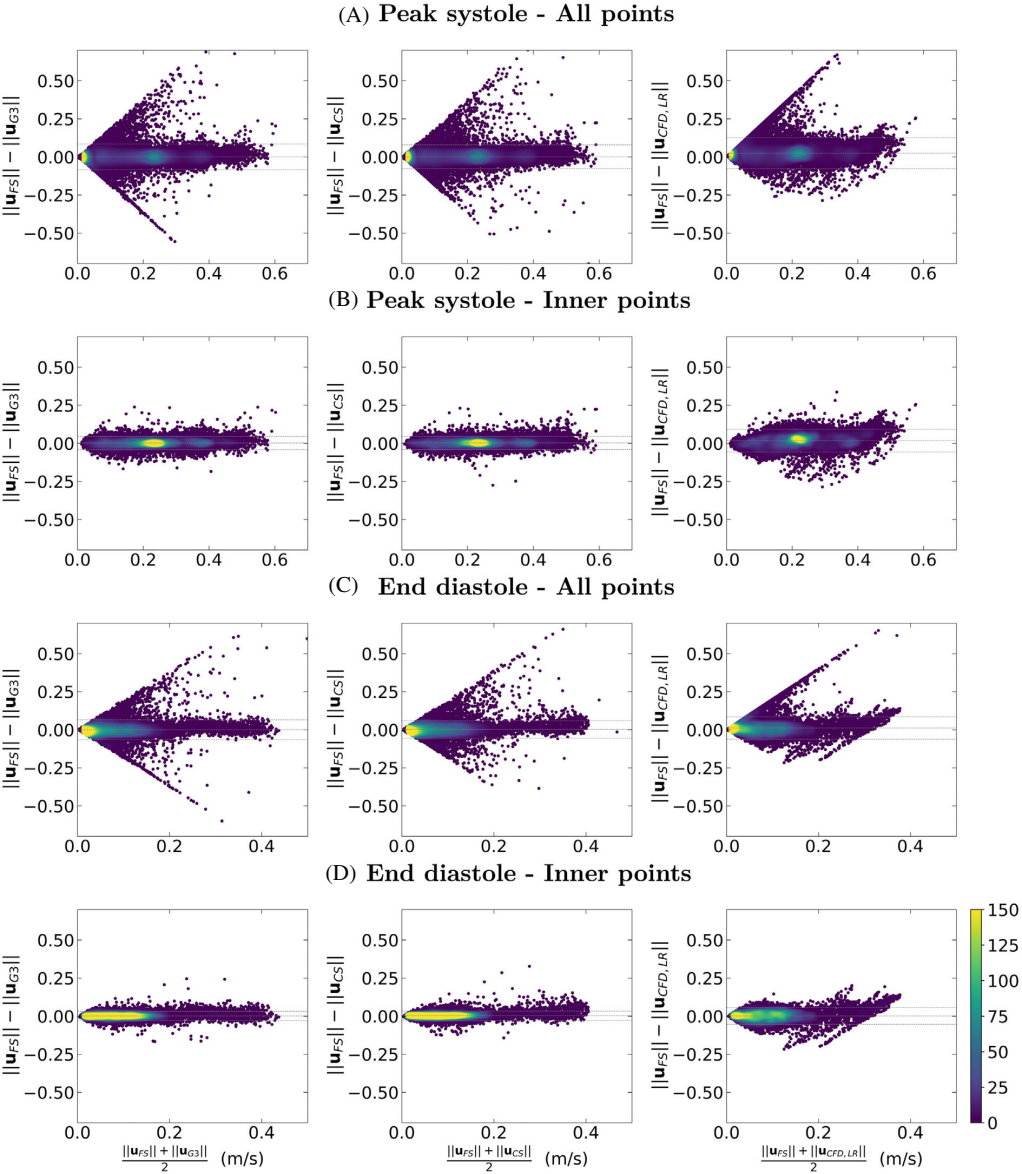
Bland–Altman plots for analyses of FS MR acquisition against G3, CS, and CFD_LR. “All points” refers to comparisons where all voxels of the phantom are included, whereas the voxels straddling the wall are removed in the “inner points” comparisons.

A Bland Altman analysis is performed to assess the velocity magnitude agreement between FS acquisition and the other modalities. Results are displayed in Figure 7. It is observed that the voxels straddling the phantom wall are responsible for the most part of the velocity dispersion for all sequences and time points. Indeed, when comparing 4D flow acquisitions 2 by 2 with all voxels, the maximal errors on velocities form linear patterns seen on both sides of the bias in the plots. The voxels forming these lines correspond to the limit case, where 1 of the velocity magnitudes is almost zero (as expected close to the wall), whereas the other is not, producing the slope of ±2. One can observe that this line is only seen in the upper part of the plot for the comparison against CFD_LR. This is because of the noise‐free high‐resolution CFD, given that the downsampling process consists in interpolating the high‐resolution CFD velocity field onto a subdivision of the MRI grid and to average the velocities of the subvoxels present within each voxel of the MRI grid. Thereby, a voxel straddling the phantom wall will have a CFD_LR velocity, which is an average of velocities for subvoxels within the phantom and zeros for subvoxels outside. In contrast, an MR edge voxel is capturing isochromats velocities and noise.

Once these edge voxels are taken out of the comparison, good agreement is found with low bias and narrow 95% confidence interval. In this latter comparison, the velocity magnitude difference (reported as bias ±1.96 SD, in [m/s]) between FS and, respectively, G3, CS, and CFD_LR, are found to be 0.00 ± 0.04, 0.00 ± 0.04, and 0.02 ± 0.08 at peak systole and 0.00 ± 0.03, 0.00 ± 0.03, and 0.00 ± 0.06 at end diastole. It has yet been noticed that whereas the velocity scattering is rather symmetrical for the comparison with the accelerated MR sequences, there is some shift toward higher velocity magnitudes for FS as compared to CFD_LR.

**FIGURE 8:**
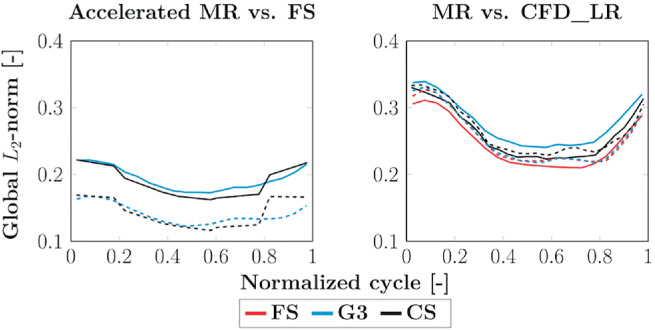
Global $L_{2}$‐norm of the velocity vector differences along the cardiac cycle. On the left, the accelerated MR sequences are compared to the FS MR acquisition. On the right, the MR sequences are compared to CFD_LR. The solid lines refer to the average over all voxels of the segmented volume, whereas the dashed ones correspond to the average over the voxels strictly included inside the phantom segmentation.

To have an overview of the global error distribution over time, the $L_{2}$‐norm over all voxels is computed and presented in Figure 8. Good agreements are found with respect to the FS acquisition, with an average $L_{2}$‐norm [unitless] decreasing from 0.193 to 0.141 for G3 and from 0.188 to 0.143 for CS when removing the edge voxels. When comparing the MR data with CFD_LR, the error increases from 0.248 to 0.254 for FS and from 0.262 to 0.266 for CS, whereas there is a decrease from 0.277 to 0.255 for G3 when taking the edge voxels out of the norm computation. For reference, the average $L_{2}$‐norm when comparing CFD_LR with CFD_HR is 0.022 and 0.018 [−], respectively, with and without the edge voxels.

### 3.3 Flow rates and peak velocities

**FIGURE 9:**
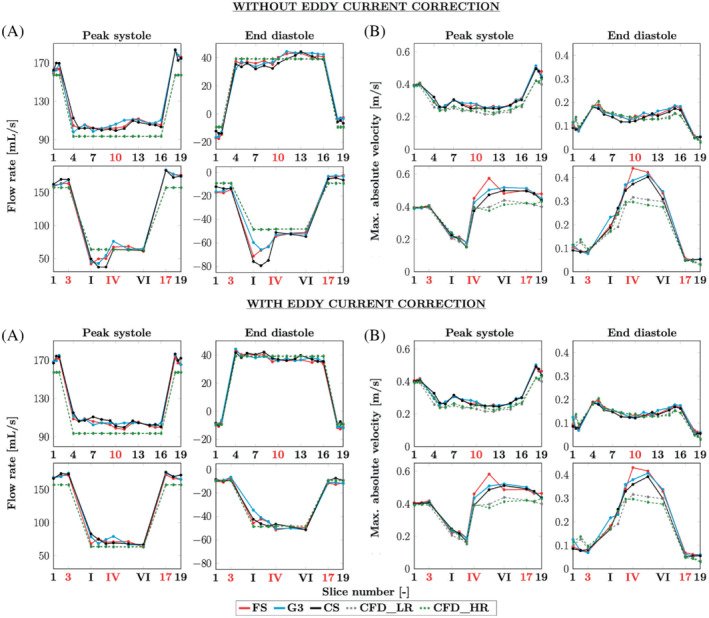
Flow rates (computed on all voxels) and peak velocities (computed on inner voxels only) for the velocity fields before (on the top) and after eddy current correction (at the bottom). Both are divided in (A) flow rates and (B) peak velocities at peak systole and end diastole. Above: along the main duct from planes 1 to 19. Below: along the collateral duct, from planes 1–3, I–VI, and 17–19, as referenced in Figure 3. The slice numbers in red indicate the planes, which were highlighted in Figure 3 and are only meant to make the plots more readable.

Volumetric flow rates and peak velocities are presented in Figure 9 A, B, respectively. These quantities are presented for the MR velocity fields before and after eddy currents correction to highlight how this correction acts on the data. The patterns observed for the 3 MR modalities are globally quite similar. The eddy currents correction helps in regularizing the measured flow rates throughout the phantom, thereby better complying with the principle of mass conservation (note that the CFD method used here is designed to meet this principle).

Indeed, the flow rates observed at the inlet and at the outlet are more consistent with each other when the effect of the eddy currents is removed. Also note the drop in the aneurysm‐like region (between planes III and IV), which disappears when the correction is applied, leading to better agreement between the measurements along the collateral. At peak systole, the flow rates observed in the main duct tend to be overestimated by the MR acquisitions with respect to the CFD simulation. Concerning the peak velocities, although those from the CFD are globally smaller than those from the MRI, similar patterns are observed. Note that to avoid any noise due to partial volume effects at the phantom wall, the peak velocities are computed only on inner voxels, as for the Bland–Altman plots in Figure 7. Yet, it has been reported that keeping the edge voxels improves the flow rates measurements.^38^ Thus, these voxels are included to compute the flow rates displayed in Figure 9.

## 4 DISCUSSION

The aim of this study is to evaluate several acceleration techniques of 4D flow MRI, namely GRAPPA *R* = 2, 3, 4 and prototypal CS *R* = 7.6 sequences, against gold‐standard FS k‐space as well as CFD simulations. In order to compare all acquisitions under the same well‐controlled conditions, a rigid flow phantom is used in vitro and simulated in silico. Such a setup presents many advantages as compared to in vivo situations because it removes some sources of uncertainties associated with wall motion, segmentation errors, and blood rheology. Th usual postprocessing (Maxwell terms, distortions, noise masking, phase unwrapping, and eddy currents correction) is applied to MR images, and the CFD data is phase‐averaged and downsampled toward the MRI resolution to enable comparison on the same grid.

Qualitatively, all modalities show good visual velocity agreement along in‐plane directions x and z. However, the through‐plane (y) velocity v appears to be less replicable from a modality to another. Low velocity‐to‐noise ratio related to generally low v velocities^39^ as well as high flow fluctuations could be responsible for these discrepancies.

Quantitatively, the good agreement between both CS and G3 accelerated MRI techniques and the FS acquisition is further confirmed in the Bland–Altman and global $L_{2}$‐norm plots. For both indicators, a better agreement is found when the voxels located at the phantom walls are removed. The velocities recorded at these points suffer from a poor velocity‐to‐noise ratio due to both low velocities and low SNR at the interface. Indeed, the complex signals recorded at voxels straddling the edge include random velocity variations between ±VENC due to the plastic phantom walls.

Even though the FS, G3, and CS acquisitions globally agree with each other, some local discrepancies have been noticed. One explanation could be the SNR, which varies depending on the chosen modality. Jung et al.^14^ reported higher SNR for Parallel MRI with extended and averaged GRAPPA kernels (PEAK‐GRAPPA) as compared to conventional GRAPPA, due to the intrinsic temporal averaging properties of the first method, which is based on the k‐t GRAPPA technique. Because the CS sequence used in this study is also based on a k‐t accelerated method, higher SNR is expected for this acquisition, hence higher quality of the PC images. Another ground for the differences between the MR images could arise from the reconstruction framework. Both FS and G3 are reconstructed using the scanner's adaptive combination method,^40^ which according to Ros et al.^41^ leads to signal loss in magnitude images and errors in phase determination. Furthermore, shortening TE has been shown to compensate for higher‐order motion encoding,^42^ and a shorter TE is used for the CS acquisition. Signal loss is also visually noticed for all MR modalities at the outlet region downstream of the collateral branch, especially at peak systole (see Supporting Information Figure S2). O'Brien et al.^43^ reported signal attenuation associated with flow errors in high‐velocity turbulent jets as studied on a stenotic phantom under steady flow. They suggested that turbulence could be one of the reasons of intravoxel dephasing, leading to signal loss. The dephasing could be further amplified under pulsatile flow due to temporal accelerations.

Regarding the comparison with low‐resolution CFD, once edge voxels are taken out of the $L_{2}$‐norm, all MRI modalities present similar outcomes. However, whereas removing the edge voxels reduces the global $L_{2}$‐norm for each time frame for G3, the error increases for FS and CS for the time instants between peak systole and end diastole. One explanation of this phenomenon could again come from the averaging of edge voxels. The contribution of the random phase noise in MRI edge voxels can virtually lead to a maximal voxel‐wise $L_{2}$‐error comprised between [−2 VENC, 2 VENC] when comparing 2 MRI modalities with each other and between [−VENC, VENC] when comparing MRI with CFD. Thereby, lower error levels are expected in these voxels in the comparison with respect to CFD. It appears that for FS and CS, the contribution of the edge voxels to the global $L_{2}$‐norm is lower than the errors arising from the higher velocities found in the inner voxels.

Some discrepancies are also observed between MRI and CFD_LR velocity profiles, notably in the collateral duct and around the junction with the descending main pipe. Concerning peak velocities, they tend to be overestimated by all MRI techniques in regions and time instants associated with high velocity, such as in the main duct at peak systole; or with high acceleration, such as in the narrowing collateral duct (cross‐sections IV‐VI). These deviations could be related to velocity‐ and acceleration‐induced displacement artifacts.^44^ In 4D flow MRI, a spatial misregistration arises when the spins move during the spatial encoding along the different directions. Similarly, velocity‐displacement artifacts are induced by acceleration of the spins during the 3 velocity encodings. Regarding the eddy current correction, it does not appear to affect the trend observed for the peak velocity measurements. Nevertheless, this correction improves the flow rates for all modalities. Because the eddy current correction acts everywhere, it benefits the aneurysm‐like region in which cross‐sections are wider and velocity levels lower; in that region, the improvement of the flow rate assessment is spectacular. Yet, the overall good agreement between MRI and low‐resolution CFD for flow rates could also result from compensations of the errors arising from various artifacts (e.g., spatial misregistration, partial volume effects), which do not affect the velocity in the same manner depending on voxel locations. CFD limitations are other sources of differences in the comparisons. A first limitation comes from the boundary condition at the inlet, which is prescribed from experimental data. To study the sensitivity of the inflow waveform onto the predicted flow field, additional CFD simulations have been conducted with the inlet velocity imposed by a 2D cine PC‐MRI scan with both a finer voxel size (0.8 × 0.8 × 6 mm vs. 2 × 2 × 2 mm) and higher temporal resolution (30 reconstructed cardiac phases vs. 20 for the FS acquisition). The 2D cine PC‐MRI was acquired during the same protocol as the 4D flow MRI acquisitions, during which the pulsatile flow rate over time was controlled using an ultrasonic flowmeter. Although the 2D cine PC‐MRI could not be corrected for eddy currents due to a lack of static tissues in the thick acquired slice, no significant differences have been observed between the CFD simulations computed using this experimental acquisition or the 4D flow ones. Figures and videos presenting these comparisons are available as Supporting Information (Supporting Information Figures S3 and S4) (Supporting Information Videos S7 to S12). Nevertheless, both the 4D flow acquisitions and the 2D cine PC‐MRI scans were acquired with the magnet isocenter centered above the collateral duct. Thus, the inlet boundary of the phantom is more prone to geometric distortion and errors in velocity encoding.^45^ Another limitation of our CFD simulations consists of the time instants when the comparisons are made, which could result in a temporal shift between the MRI acquisitions and the simulations. For each cardiac phase, the 3 velocity directions are sequentially encoded in the PC‐MRI acquisition, whereas the simulation displays the velocity vector at the middle of this encoding time window. In particular, the k‐space encoding pattern in our acquisitions was the following: flow‐compensated reference, *y*‐direction, *x*‐direction, and *z*‐direction. This could potentially explain the flow structures observed in Figure 5, where the MRI patterns seem to be in advance as compared to the CFD, as well as the higher flow rates recorded along the main pipe at peak systole (Figure 9).

As reported by Ma et al.^12^ in their flow phantom, good visual agreement and voxel‐wise comparison are observed between the CS and conventional 4D flow. Even if some underestimations of the peak velocities by CS with respect to G3 can be noticed in the collateral duct, CS is not clearly found to underestimate the flow rates. An explanation for this discrepancy between the 2 studies could be the flow phantom and its circuit. The present study is a simplified setup in comparison to the more complex in vivo cardiovascular conditions. By contrast, the phantom developed by Ma et al. was based on the aorta of a healthy subject. Furthermore, the flow was controlled by a pneumatically driven ventricular assist device and pump control unit, whereas a programmable pump is used in the present work. Finally, a realistic aorta pulsatile flow was generated by Ma et al., whereas a sinusoidal flow is investigated in this study. Prescribing an aorta‐like inflow waveform, as well as accounting for the blood vessel compliance, could be first steps toward introducing more realistic flow patterns in our phantom circuit. However, the latter would require either knowledge of the wall location during the cardiac cycle or of the mechanical properties of the vessel wall to perform fluid–structure interaction CFD simulations. Also, blood behaves as a non‐Newtonian fluid, which makes its rheology more complex than the assumed Newtonian blood‐mimicking fluid used in this setup.

In this study, artifacts inherent to all MR modalities have been highlighted. Future work could include implementing postprocessing methods to compensate for artifacts on velocity fields due to acceleration‐induced displacement^46^ and to gradient field distortions.^45^ Another perspective is to simulate 4D flow MRI to further characterize the observed divergences,^47^ for instance by considering the Rician distribution of the noise in the magnitude images in MRI^48^ or computing acceleration‐induced displacement artifacts.^49^

## 5 CONCLUSION

This study demonstrated under in vitro conditions that the highly accelerated CS 4D flow MRI at *R* = 7.6 shows good agreement with the nonaccelerated FS acquisition as well as with conventional GRAPPA. However, all modalities suffered from artifacts inherent to the PC acquisition procedure. Further investigations could be carried on in more physiological conditions. Moreover, CFD simulations are a tool of interest to investigate the observed discrepancies, even though it also presents some limitations and care should be taken in modeling the investigated problem.

## CONFLICT OF INTEREST

The study was funded by Spin Up. Morgane Garreau and Thomas Puiseux are paid employees of Spin Up; Ramiro Moreno is paid employee of ALARA Expertise; and Solenn Toupin and Daniel Giese are paid employees of Siemens Healthcare. Funders and employers did not have any role in the study design, data collection and analysis, decision to publish, or preparation of the manuscript.

## Supporting information


**FIGURE S1:** Global $L_{2}$‐norm of the velocity vector differences along the cardiac cycle for all MR acquisitions. On the left, the accelerated MR sequences are compared to the fully sampled MR acquisition. On the right, the MR sequences are compared to CFD_LR. The solid lines refer to the average over all voxels of the segmented volume, whereas the dashed ones correspond to the average over the voxels strictly included inside the phantom segmentation.
**FIGURE S2:** Image magnitude before any correction at peak systole (above) and end diastole (below) for the fully sampled, GRAPPA 3 and compressed sensing 4D flow MRI acquisitions.
**FIGURE S3:** Velocity profiles along lines located in the coronal plane passing through the middle of the phantom. The velocity displayed corresponds to the projection onto the normal of the planes perpendicular to the ducts, referenced as in Figure 3. It is the analogous plot of Figure 6, where the curves from the additional downsampled CFD simulations based on a 2D PC‐MRI have been added (CFD_LR_FROM_2D). The former curves are kept for reference.
**FIGURE S4:** Flow rates (computed on all voxels) and peak velocities (computed on inner voxels only) for the velocity fields after eddy current correction, divided in (A) Flow rates and (B) Peak velocities at peak systole and end diastole. *Above*: along the main duct from planes 1–19. *Below*: along the collateral duct, from planes 1–3, I–VI and 17–19, as referenced in Figure 3. It is the analogous plot of Figure 9, where the curves from the additional downsampled CFD simulations based on a 2D PC‐MRI have been added (CFD_LR_FROM_2D). The former curves are kept for reference.

**VIDEO S1:** 3D vector‐based visualization of the fully sampled (FS) acquisition and its corresponding CFD simulations (high‐resolution CFD_HR and downsampled CFD_LR velocity fields) in the whole phantom.

**VIDEO S2:** 3D vector‐based visualization of the GRAPPA *R* = 3 (G3) acquisition and its corresponding CFD simulations (high‐resolution CFD_HR and downsampled CFD_LR velocity fields) in the whole phantom.

**VIDEO S3:** 3D vector‐based visualization of the compressed sensing (CS) acquisition and its corresponding CFD simulations (high‐resolution CFD_HR and downsampled CFD_LR velocity fields) in the whole phantom.

**VIDEO S4:** 3D vector‐based visualization of the fully sampled (FS) acquisition and its corresponding CFD simulations (high‐resolution CFD_HR and downsampled CFD_LR velocity fields) in a slice in the middle of the aneurysm‐like region.

**VIDEO S5:** 3D vector‐based visualization of the GRAPPA *R* = 3 (G3) acquisition and its corresponding CFD simulations (high‐resolution CFD_HR and downsampled CFD_LR velocity fields) in a slice in the middle of the aneurysm‐like region.

**VIDEO S6:** 3D vector‐based visualization of the compressed sensing (CS) acquisition and its corresponding CFD simulations (high‐resolution CFD_HR and downsampled CFD_LR velocity fields) in a slice in the middle of the aneurysm‐like region.

**VIDEO S7:** 3D vector‐based visualization of the fully sampled (FS) acquisition and its corresponding high‐resolution CFD simulations based respectively on a 4D flow MRI (CFD_FROM_4D) and on a 2D cine PC‐MRI (CFD_FROM_2D) in the whole phantom.

**VIDEO S8:** 3D vector‐based visualization of the GRAPPA *R* = 3 (G3) acquisition and its corresponding high‐resolution CFD simulations based respectively on a 4D flow MRI (CFD_FROM_4D) and on a 2D cine PC‐MRI (CFD_FROM_2D) in the whole phantom.

**VIDEO S9:** 3D vector‐based visualization of the compressed sensing (CS) acquisition and its corresponding high‐resolution CFD simulations based respectively on a 4D flow MRI (CFD_FROM_4D) and on a 2D cine PC‐MRI (CFD_FROM_2D) in the whole phantom.

**VIDEO S10:** 3D vector‐based visualization of the fully sampled (FS) acquisition and its corresponding high‐resolution CFD simulations based respectively on a 4D flow MRI (CFD_FROM_4D) and on a 2D cine PC‐MRI (CFD_FROM_2D) in a slice in the middle of the aneurysm‐like region.

**VIDEO S11:** 3D vector‐based visualization of the GRAPPA *R* = 3 (G3) acquisition and its corresponding high‐resolution CFD simulations based respectively on a 4D flow MRI (CFD_FROM_4D) and on a 2D cine PC‐MRI (CFD_FROM_2D) in a slice in the middle of the aneurysm‐like region.

**VIDEO S12:** 3D vector‐based visualization of the compressed sensing (CS) acquisition and its corresponding high‐resolution CFD simulations based respectively on a 4D flow MRI (CFD_FROM_4D) and on a 2D cine PC‐MRI (CFD_FROM_2D) in a slice in the middle of the aneurysm‐like region.
