# Supplementary material for: Accelerated sequences of 4D flow MRI using GRAPPA and compressed sensing: A comparison against conventional MRI and computational fluid dynamics
Source: Magn Reson Med. 2022 Aug 25;88(6):2432–46. doi: 10.1002/mrm.29404 (PMC9804839; doi:10.1002/mrm.29404)
Supplement: Supplementary file 1 — FIGURE S1: Global L2‐norm of the velocity vector differences along the cardiac cycle for all MR acquisitions. On the left, the accelerated MR sequences are compared to the fully sampled MR acquisition. On the right, the MR sequences are compared to CFD_LR. The solid lines refer to the average over all voxels of the segmented volume, whereas the dashed ones correspond to the average over the voxels strictly included inside the phantom segmentation. FIGURE S2: Image magnitude before any correction at peak systole (above) and end diastole (below) for the fully sampled, GRAPPA 3 and compressed sensing 4D flow MRI acquisitions. FIGURE S3: Velocity profiles along lines located in the coronal plane passing through the middle of the phantom. The velocity displayed corresponds to the projection onto the normal of the planes perpendicular to the ducts, referenced as in Figure 3. It is the analogous plot of Figure 6, where the curves from the additional downsampled CFD simulations based on a 2D PC‐MRI have been added (CFD_LR_FROM_2D). The former curves are kept for reference. FIGURE S4: Flow rates (computed on all voxels) and peak velocities (computed on inner voxels only) for the velocity fields after eddy current correction, divided in (A) Flow rates and (B) Peak velocities at peak systole and end diastole. Above: along the main duct from planes 1–19. Below: along the collateral duct, from planes 1–3, I–VI and 17–19, as referenced in Figure 3. It is the analogous plot of Figure 9, where the curves from the additional downsampled CFD simulations based on a 2D PC‐MRI have been added (CFD_LR_FROM_2D). The former curves are kept for reference. [file MRM-88-2432-s009.pdf]

SUPPORTING INFORMATION - FIGURES

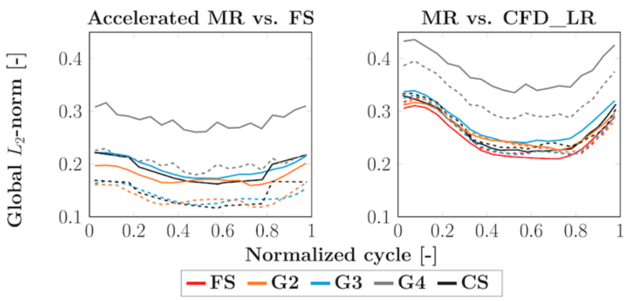

Figure S1: Global L_2-norm of the velocity vector differences along the cardiac cycle for all MR acquisitions. On the left, the accelerated MR sequences are compared to the fully sampled MR acquisition. On the right, the MR sequences are compared to CFD_LR. The solid lines refer to the average over all voxels of the segmented volume, whereas the dashed ones correspond to the average over the voxels strictly included inside the phantom segmentation.

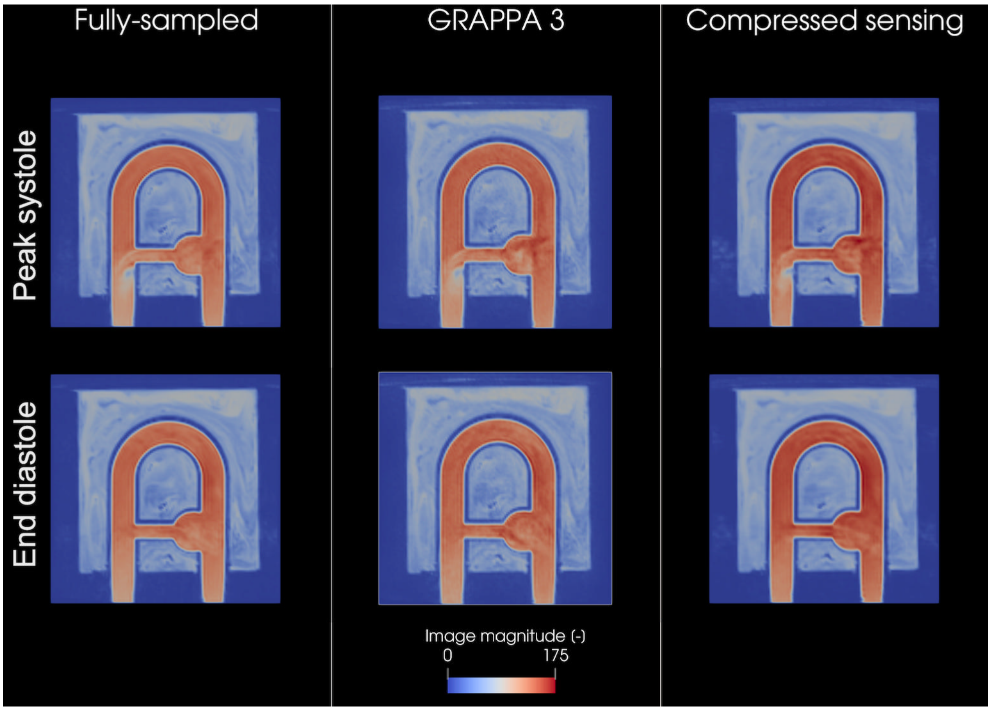

Figure S2: Image magnitude before any correction at peak systole (above) and end diastole (below) for the fully sampled, GRAPPA 3 and compressed sensing 4D flow MRI acquisitions.

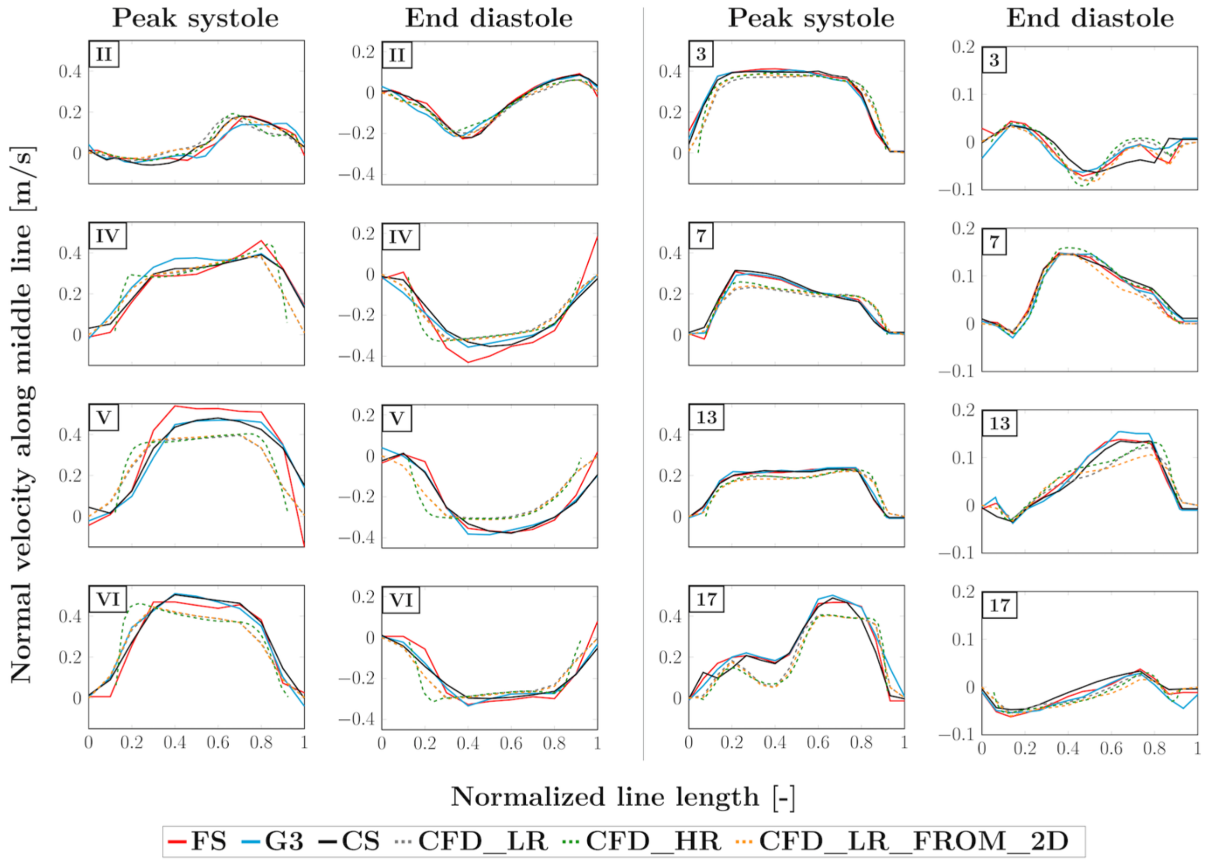

Figure S3: Velocity profiles along lines located in the coronal plane passing through the middle of the phantom. The velocity displayed corresponds to the projection onto the normal of the planes perpendicular to the ducts, referenced as in Figure 3. It is the analogous plot of Figure 6, where the curves from the additional downsampled CFD simulations based on a 2D PC-MRI have been added (CFD_LR_FROM_2D). The former curves are kept for reference.

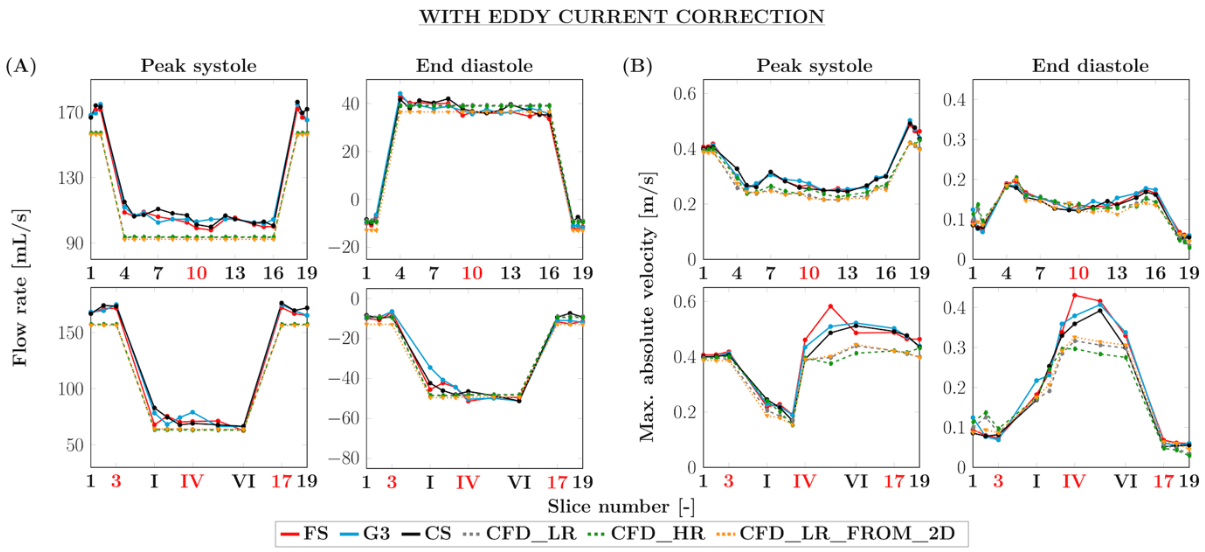

Figure S4: Flow rates (computed on all voxels) and peak velocities (computed on inner voxels only) for the velocity fields after eddy current correction, divided in (A) Flow rates and (B) Peak velocities at peak systole and end diastole. ***Above:*** *along the main duct from planes 1 to 19.* ***Below:*** *along the collateral duct, from planes 1-3, I-VI and 17-19, as referenced in Figure 3. It is the analogous plot of Figure 9, where the curves from the additional downsampled CFD simulations based on a 2D PC-MRI have been added (CFD_LR_FROM_2D). The former curves are kept for reference.*
